# Genetic Diversity and Structure of *Sinopodophyllum hexandrum* (Royle) Ying in the Qinling Mountains, China

**DOI:** 10.1371/journal.pone.0110500

**Published:** 2014-10-15

**Authors:** Wei Liu, Dongxue Yin, Jianjun Liu, Na Li

**Affiliations:** College of Forestry, Northwest A & F University, Yangling, China; Institute for Sustainable Plant Protection, C.N.R., Italy

## Abstract

*Sinopodophyllum hexandrum* is an important medicinal plant whose genetic diversity must be conserved because it is endangered. The Qinling Mts. are a *S. hexandrum* distribution area that has unique environmental features that highly affect the evolution of the species. To provide the reference data for evolutionary and conservation studies, the genetic diversity and population structure of *S. hexandrum* in its overall natural distribution areas in the Qinling Mts. were investigated through inter-simple sequence repeats analysis of 32 natural populations. The 11 selected primers generated a total of 135 polymorphic bands. *S. hexandrum* genetic diversity was low within populations (average H_e_ = 0.0621), but higher at the species level (H_e_ = 0.1434). Clear structure and high genetic differentiation among populations were detected by using the unweighted pair group method for arithmetic averages, principle coordinate analysis and Bayesian clustering. The clustering approaches supported a division of the 32 populations into three major groups, for which analysis of molecular variance confirmed significant variation (63.27%) among populations. The genetic differentiation may have been attributed to the limited gene flow (N_m_ = 0.3587) in the species. Isolation by distance among populations was determined by comparing genetic distance versus geographic distance by using the Mantel test. Result was insignificant (*r* = 0.212, *P* = 0.287) at 0.05, showing that their spatial pattern and geographic locations are not correlated. Given the low within-population genetic diversity, high differentiation among populations and the increasing anthropogenic pressure on the species, in situ conservation measures were recommended to preserve *S. hexandrum* in Qinling Mts., and other populations must be sampled to retain as much genetic diversity of the species to achieve ex situ preservation as a supplement to in situ conservation.

## Introduction

*Sinopodophyllum hexandrum* (Royle) Ying, family Berberidaceae, the only species of this genus in China, commonly known as Himalayan mayapple, is an endangered and medicinal perennial herb native to the Himalayan regions at elevations ranging from 2 700 m to 4 500 m [1]–[4]. Plants provide us with many important medicaments, including anticancer and antiinfective agents [5], and traditional Chinese medicine has contributed to identifying these substances [6]. *S. hexandrum* is a traditional Chinese medicine that has been used in folk medicine [7]. The roots and rhizomes of *S. hexandrum* contain large amounts of lignans. The most important lignan for human health is arguably the most active cytotoxic aryltetralin lignan, podophyllotoxin, with three times the podophyllotoxin levels compared to the American species *Podophyllum peltatum* [8], [9], [10], as a precursor for the semi-synthesis of the anticancer pharmaceuticals, such as etoposide (VP-16), teniposide (VM-26), GP-7, NK-611, etopophos, GL-331 and TOP-53 [9]–[15]. The destructive harvest of these plants added *S. hexandrum* to the endangered species list of the Convention on International Trade in Endangered Species of Wild Fauna and Flora [16]. *S. hexandrum* was classified as an endangered species (grade 3) in 1987 by the Chinese Plant Red Book [4].

Currently, with the enhanced awareness of its medicinal value and superior efficacy in clinical applications, the wild *S. hexandrum* populations in China has been noted to be very small and to be rapidly declining. The availability of podophyllotoxin from plants has become increasingly limited due to intense collection, habitat fragmentation, low natural regeneration rate, and the lack of organized cultivation. Wild *S. hexandrum* populations could become extinct without timely and effective protective measures, costing humans an ideal drug against cancer. Knowledge of the genetic diversity at intraspecific levels is an important prerequisite for species conservation and a rational exploitation program planning. However, previous studies have mainly focused on the identification and separation of the chemical components [17]–[25], biological properties [26]–[32], and micropropagation of *S. hexandrum* [33]. Only studies on genetic diversity in *S. hexandrum* from the Northwestern Himalayan region are available [34], [35], [36]. In particular, two areas where *S. hexandrum* is grown, namely, western Sichuan Province and Himalaya–Hengduan Mt. region in China, were investigated to identify genetic diversity of this species through inter-simple sequence repeats (ISSR) and amplified fragment length polymorphism (AFLP) markers [37], [38], [39], respectively. These reports with same results showed that *S. hexandrum* populations had relatively high genetic diversity (H_e_ = 0.2944∼0.3377). However, there is a consensus that alpine plants are faced with pollinator restriction [40]. The unclear extent of the species dispersal mechanisms makes it interesting to study the relationships between populations. Furthermore, many reports about genetic diversity of other medicinal plants, such as *Calamagrostis porteri ssp. insperata* [41], *Aegiceras corniculatum* [42], *Sonneratia alba* [43], *Coscinium fenestratum* [44], *and Lilium pumilum* [45], have been published based on the ISSR approach.

Molecular markers are very useful tools for genetic diversity studies. ISSR markers are molecular markers especially suited to genetic polymorphisms analyses of species without available sequence information [46], [47]. Studies on the population relationships, genetic diversity and conservation of *S. hexandrum* in Qinling Mts. are requisite because climate change and local overexploitation may cause unknown endangering mechanisms. The present study aims to establish management strategies for the conservation genetics of *S. hexandrum* by (1) examining the levels of genetic variability within and among *S. hexandrum* populations sampled from Qinling Mts. where historical records showed *S. hexandrum* having grown naturally, (2) assessing the possible factors that affect the genetic variation observed, and (3) comparing these within and among *S. hexandrum* populations with data published for itself or other plant taxa with similar characteristics.

## Materials and Methods

### Ethics statement

The endangered species were collected, and research activities were scientifically conducted under the permits issued by the local forestry department. A detailed description of the experimental material collection and procedures is provided. The experimental procedures were approved by the Ethics Committee for Plant Experiments of Northwest A & F University and the State Forestry Administration, P. R. China. The names of the authorities that issued the permit for each location were listed in Table S1 in Text S1.

### Study area

**Figure 1:**
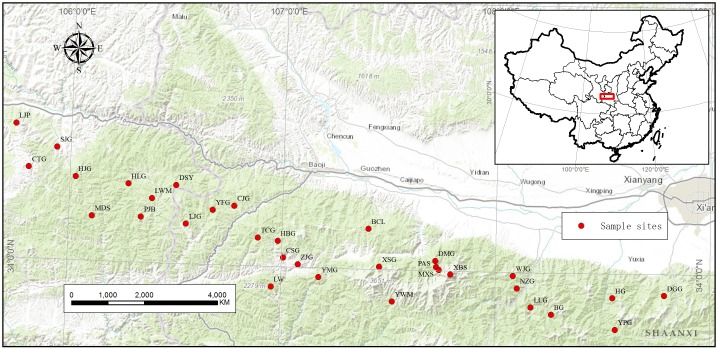
Locations of the 32 *S. hexandrum* populations in Qinling Mts. sampled for this study.

This study was performed in the Qinling Mts. (32°41′ to 34°59′ N, 103°54′ to 110°34′ E), which are located in central of China (Figure 1). The Qinling Mts., a 1 500 Km mountain chain, run east–west and act as an important watershed divider between two great Chinese rivers, the Yangtze River and the Yellow River, which constitute a transitional zone between the northern subtropical zone and warm-temperate zone. The Qinling Mts. were considered to be a biodiversity hotspot in China [48]. As one of the distribution areas of *S. hexandrum*, Qinling Mts. have unique environmental features which have high impact on the evolution of the species.

### Plant materials

*S. hexandrum* distribution pattern and extent of in Qinling Mts. were investigated from 2010 to 2011. The *S. hexandrum* populations are distributed in small and scattered patches. According to the field survey information, a total of 32 wild *S. hexandrum* populations were sampled for DNA analysis between July 19, 2012, and September 17, 2012, to ensure collection period consistency (Table 1). *S. hexandrum* has a wide geographic distribution throughout the Qinling Mts. (Figure 1). The altitude of the sample sites ranged from 1 013 m to 2 883 m (Table1). Geographical distances between populations ranged from 5.5 km to 276.8 km. 20 plants were sampled from each population. The horizontal and vertical distances between sampled plants within each population were over 20 and 5 m, respectively, to increase the likelihood of sampling inter-individual variation within each population [37], [38]. About 2 g to 10 g of fresh young leaves per plant was immediately frozen in liquid nitrogen and then kept at −80 °C until DNA isolation. The key information on *S. hexandrum* populations in all sampling sites is summarized in Table 1.

**Table 1:** Sample information of the 32 sampling sites.

No.	Population	Prefecture	Coordinates	N	Altitude/m	SO/SD/°	Vegetation type
1	Caotangou	Maiji	E105°48′N34°21′	20	1 899	SE70	Shrub-grass
2	Tancaogou	Fengxian	E106°53′N34°6′	20	1 728	SW45	Shrub-grass
3	Chunshugou	Fengxian	E107°1′N34°2′	20	1 512	SW65	Shrub-grass
4	Hougou	Fengxian	E108°33′N33°55′	20	1 439	N75	Shrub-grass
5	Xiaoshagou	Taibai	E107°27′N34°1′	20	2 332	NW60	Forest-edge, Shrub-grass
6	Doumugong	Meixian	E107°43′N34°2′	20	2 748	NW75	Forest-edge, Shrub-grass
7	Pinganssi	Meixian	E107°43′N34°1′	20	2 815	NW75	Forest-edge, Shrub-grass
8	Mingxingsi	Meixian	E107°44′N34°0′	20	2 637	E75	Forest-edge, Shrub-grass
9	Xiabansi	Meixian	E107°47′N33°59′	20	2 883	SE50	Forest-edge, Shrub-grass
10	Laojungou	Liangdang	E106°33′N34°9′	20	1 902	SE60	Cliff, edge of forest
11	Youfanggou	Fengxian	E106°40′N34°12′	20	1 545	SW70	Shrub-grass
12	Baicaoling	Taibai	E107°24′N34°9′	20	1 373	S60	Forest-edge, Shrub-grass
13	Nianzigou	Zhouzhi	E108°6′N33°56′	20	1 821	SW75	Meadow, edge of forest
14	Wenjiagou	Zhouzhi	E108°5′N33°59′	20	1 765	S80	Meadow, edge of forest
15	Liulingou	Zhouzhi	E108°10′N33°52′	20	1 013	NW75	Meadow, edge of forest
16	Beigou	Zhouzhi	E108°16′N33°51′	20	1 579	NW85	Meadow, edge of forest
17	Dagangou	Huxian	E108°47′N33°55′	20	1 335	NW65	Shrub-grass
18	Yaowangmiao	Taibai	E107°31′N33°53′	20	1 779	SE40	Forest-edge, Shrub-grass
19	Yingpangoukou	Huxian	E108°34′N33°47′	20	1 487	SE60	Shrub-grass
20	Maiduoshigou	Liangdang	E106°7′N34°10′	20	1 520	SE70	Cliff, edge of forest
21	Panjiaba	Liangdang	E106°20′N34°10′	20	1 976	SW45	Cliff, edge of forest
22	Zhangjiagou	Fengxian	E107°5′N34°1′	20	1 469	SW75	Shrub-grass
23	Longwangmiao	Fengxian	E106°57′N33°55′	20	1 475	SW75	Shrub-grass
24	Huangbaigou	Fengxian	E106°59′N34°6′	20	1 745	SW80	Shrub-grass
25	Dashuiyugou	Maiji	E106°30′N34°18′	20	1 878	W75	Shrub-grass
26	Chenjiagou	Fengxian	E106°46′N34°13′	20	1 515	SW45	Shrub-grass
27	Longwangmiao	Maiji	E106°23′N34°15′	20	1 736	NW60	Shrub-grass
28	Yinmagou	Taibai	E107°11′N33°58′	20	2 252	S60	Forest-edge, Shrub-grass
29	Hualingou	Maiji	E106°17′N34°18′	20	1 793	SW55	Shrub-grass
30	Huojigou	Maiji	E106°2′N34°19′	20	1 718	SE65	Shrub-grass
31	Shijiagoucun	Maiji	E105°56′N34°25′	20	1 366	SE60	Shrub-grass
32	Liujiaping	Maiji	E105°44′N34°30′	20	1 690	SW30	Shrub-grass

**Note**: N means sample size; SO/SD means slope orientation/degree.

### DNA extraction

Total genomic DNA was extracted from frozen leaves by using a plant genomic DNA rapid extraction kit (Spin-column) (BioTek Corporation, Beijing, China; http://bioteke.biogo.net/). The extracted DNA was quantified by comparing with known DNA of standard quantity (Lambda DNA) through electrophoresis in ethidium bromide-stained 1.0% agarose gels (Gene Genius Bio Imaging System; Synegene), and the extracted DNA was diluted in TE buffer to a final concentration of 50 ng/µL and stored at−20°C before PCR amplification.

### Primer screening and ISSR-PCR amplification

A total of 100 ISSR primers (synthesized by Shanghai Sheng Gong Biotechnology CO. LTD, China) were screened based on the primer set published by the Biotechnology Laboratory, University of British Columbia, Canada (UBC set No. 9) and the studies on Himalayan mayapple [34]–[39]. An optimum reaction system was obtained by screening DNA, Mg^2+^, dNTP, primer (UBC900 was used for preliminary test), and Taq DNA polymerase concentrations and annealing temperature, and reaction conditions. The optimization showed that 20 µL of reaction system is ideal. Each 20 µL amplification reaction consisted of 1×PCR buffer (10 mM Tris-HCl at pH 8.3, 50 mM L^–1^ KCl, 0.001% gelatin, and l.5 mmol L^–1 ^MgCl_2_), 1.6 mmol L^–1^ dNTP mix, 0.6 µmol L^–1^ primer (UBC900 was used for preliminary test), 15 ng of template DNA, and 1.0 U Taq DNA polymerase (TaKaRa Biotechnology, Dalian, China), using a cycling profile of initial 5 min at 94°C, followed by 45 cycles of 30 s at 94°C, 45 s annealing at 50°C, and 90 s extension at 72°C, ending with a final extension of 7 min at 72°C.

The optimized PCR experiment conditions were applied for primer screening in a PTC100TM Programmable Thermal Controller (MJ Research, Waltham, MA, USA). Six populations (TCG, LJG, BG, MDS, YMG and CTG) with observable variations (morphology, habitat, etc) were selected to initially screen 100 primers by using 10 samples for each population. Primers that generated scorable bands and high levels of polymorphisms were selected by genotyping all populations. The amplification products were electrophoresed on 1.0% agarose gels buffered with 1.0×TBE for 2.5 h at 100 V and were detected through ethidium bromide staining, and the gels were imaged in the Gene Genius Bioimaging System. Band size was estimated from a 0.1 kbp DNA ladder (TaKaRa Biotechnology, Dalian, China). Each primer was amplified in triplicate to confirm reliability and reproducibility. A reaction without DNA was used as negative control.

### Data analyses

Amplification results were scored according to the positions of the DNA bands from electrophoresis, being labeled “1” for presence of the bands and “0” for absence of the bands in the data matrix. Only stable bands with repeatable differences were considered valid for polymorphism loci.

The resulting ISSR phenotype data matrix (binary matrix from 0 to 1) was analyzed using the POPGENE software (version 1.31) [49] to compute genetic diversity parameters, such as allele frequencies, percentage of polymorphic bands (PPB), number of alleles per locus (A_o_), effective number of alleles per locus (A_e_), total gene diversity (H_t_), the level of gene flow (N_m_), Nei’s gene diversity (H_e_), gene distance (GD), Shannon’s information index (H_o_), within-population diversity (H_s_), and mean coefficient of gene differentiation (G_st_). An unweighted pair groups mean arithmetic (UPGMA) mean dendrogram was constructed using PowerMarker 3.23 to examine the genetic relationship at the species level [50]. A bootstrap (resampling) test was performed 1 000 times to determine distances between the populations using PHYLIP version 3.69 (PHYLogeny Inference Package) programs [51]. Bayesian analysis of population structure was performed as implemented in STRUCTURE (version 2.2) to infer the most likely number of population genetic clusters (K) in the ISSR dataset [52]. K ranged from 1 to 10, with 10 replicate runs for each K, and a burn-in period of 2×10^5^ and 5×10^4^ iterations. The “no admixture model” and independent allele frequencies were chosen for this analysis. The most likely number of clusters was estimated according to the model values (ΔK) based on the second order rate of change, with respect to K, of the likelihood function [53]. To detect within-group structure, subsequent runs were performed for each obtained clusters using the same settings as previous. Population similarity was also explored and visualized through principle coordinate analysis (PCoA) using NTSYSpc 2.10e [54]. Analysis of molecular variance (AMOVA), which partitions total phenotypic variance within and among populations, was performed using WIN AMOVA (version 1.55), which was provided by the Genetics and Biometry Laboratory, University of Geneva, Geneva, Switzerland [55]. The AMOVA input files with the Euclidean distance matrix were created using AMOVA-PREP 1.01 [56]. Significance level was tested by comparing the frequency distributions from the original data and the data generated by a set of 1 000 computer simulations. A Mantel test was performed using Tools for Population Genetic Analysis (TFPGA) [56] for computing 5 000 permutations to test the isolation by distance (IBD) among populations by comparing genetic distance between all pairwise combinations of populations versus geographic distance. Geographical distance, L_ab_, was computed using the following formula: L_ab_ = Arccos [cos(LAT_a_)COS(LONG_a_)cos(LAT_b_)cos(LONG_b_) + cos(LAT_b_)sin(LONG_b_)cos(LAT_b_)sin(LONG_b_) + sin(LAT_a_)sin(LAT_b_)]×R [57]. LONG_a_, LAT_a_ and LONG_b_, LAT_b_ are the longitudes and latitudes (in radians) of sampling sites a and b, respectively; R indicates the radius of the earth, which is 6 378 km; and L_ab_ represents the geographic distance between sampling sites a and b.

## Results

### Genomic DNA amplification results

For polymorphism testing of the 640 *S. hexandrum* individuals from 32 populations, forty-eight ISSR primers amplified visible bands and were chosen from the initial set of 100 primers to screen for reproducible markers. PCR amplification results (Table 2) show that 11 primers produced 241 clear and replicated bands (250 bp to 2 000 bp), of which 135 were polymorphic (56.02%) with 100% reproducibility. Individual primers detected between 19 (UBC825) and 29 (UBC900) loci can amplify clear bands, with an average of 21.91. The percentage of polymorphism ranged from 42.86% (UBC834) to 68.97% (UBC900), indicating that the selected primers are highly polymorphic across *S. hexandrum* populations. The H_t_ was 0.1434, whereas H_s_ was found to be 0.0599. The G_st_ value of 0.5823 indicated that 41.77% of the genetic diversity resided within the populations. The N_m_ among the sampled populations was calculated as 0.3587 using G_st_ through the formula (0.5(1−G_st_)/G_st_).

**Table 2:** Description of the11 selected primers used for ISSR amplification.

No.	Primer	Sequences 5′→3′	N_t_	N_p_	P_r_ %	H_t_	H_s_	G_st_
1	UBC825	(AC)_8_T	19	12	63.16	0.1715	0.0835	0.5132
2	UBC834	(AG)_8_YT	21	9	42.86	0.1035	0.0440	0.5748
3	UBC844	(CT)_8_AGC	22	11	50.00	0.1160	0.0517	0.5543
4	UBC845	(CT)_8_AGG	23	12	52.17	0.1192	0.0494	0.5855
5	UBC853	(CT)_8_AGT	20	9	45.00	0.1121	0.0352	0.6858
6	UBC855	(AC)_8_YT	21	11	52.38	0.1347	0.0645	0.5212
7	UBC857	(AC)_8_CTG	20	13	65.00	0.1763	0.0763	0.5672
8	UBC867	(GGC)_6_	19	10	52.36	0.1251	0.0564	0.5492
9	UBC873	(GACA)_4_	22	11	50.00	0.1165	0.0441	0.6214
10	UBC895	AGAGTTGGTAGCTCTTGATC	25	17	68.00	0.1875	0.0765	0.5920
11	UBC900	ACTTCCCCACAGGTTAACACA	29	20	68.97	0.2150	0.0773	0.6404
Sum			241	135				
Mean			21.91	12.27	56.02	0.1434	0.0599	0.5823

**Note**: N_t_-No. of total amplified bands; N_p_-No. of polymorphic bands; P_r_-Polymorphism rate; H_t_- Total gene diversity among population; H_s_-within population diversity; G_st_-Mean coefficient of gene differentiation.

### Population genetic diversity

Detailed statistical analyses were performed on the ISSR amplification results (Table 3). The numbers of polymorphic bands are different among populations. The highest PPB (39.17%) was observed in the DMG population. However, only 20.73% of the bands were polymorphic in the SJG population. The PPB was 56.02% at the species level, whereas those of the single populations ranged from 20.73% (SJG) to 39.17% (DMG), with an average of 27.33%. A_e_ ranged from 1.0182 (HJG) to 1.2643 (BG), with 1.1630 at the population level and 1.3732 at the species level. H_e_ within populations was lower (0.0621) than that of the species level (0.1434). Within each population, the H_e_ of most populations ranged from 0.0226 to 0.0971. Only in the population HG, a high H_e_ (0.1229, Table 3) was observed. H_o_ ranged from 0.0244 to 0.1038, with an average of 0.0637 at the population level and 0.2362 at the species level. Allele frequencies calculated using Popgene software were shown in Table S2 in Text S2.

**Table 3:** Descriptive statistics summary of the *S. hexandrum* populations.

Population	Code	PPB (%)	A_o_	A_e_	H_e_	H_o_
Tancaogou	TCG	23.63	1.3546	1.1684	0.0559	0.0737
Chunshugou	CSG	22.62	1.2687	1.1556	0.0337	0.0689
Hougou	HG	25.66	1.5642	1.2357	0.1229	0.1038
Xiaoshagou	XSG	25.32	1.5721	1.1957	0.0777	0.0733
Doumugong	DMG	39.17	1.3905	1.1404	0.0296	0.0255
Pinganssi	PAS	33.95	1.4397	1.1833	0.0567	0.0442
Mingxingsi	MXS	26.94	1.4720	1.1667	0.0381	0.0667
Xiabansi	XBS	23.51	1.4986	1.231	0.0542	0.0614
Laojungou	LJG	28.52	1.2199	1.1937	0.0426	0.0566
Youfanggou	YFG	21.95	1.3688	1.1846	0.0533	0.0755
Baicaoling	BCL	28.84	1.5579	1.1795	0.0603	0.0715
Nianzigou	NZG	25.18	1.473	1.2166	0.0868	0.0508
Wenjiagou	WJG	31.99	1.4238	1.1737	0.0797	0.0321
Liulingou	LLG	26.85	1.5053	1.2000	0.0882	0.0733
Beigou	BG	29.01	1.5319	1.2643	0.0670	0.0680
Dagangou	DGG	27.07	1.5912	1.2128	0.0448	0.0781
Yaowangmiao	YWM	32.03	1.5309	1.2024	0.0728	0.0972
Yingpangoukou	YPG	24.93	1.6054	1.2290	0.0778	0.0799
Maiduoshigou	MDS	24.63	1.1576	1.0445	0.0226	0.0656
Panjiaba	PJB	32.95	1.1872	1.1293	0.0252	0.0277
Zhangjiagou	ZJG	26.17	1.2364	1.1722	0.0523	0.0464
Longwangmiao	LW	24.46	1.2953	1.2199	0.0498	0.0636
Huangbaigou	HBG	24.07	1.3276	1.1913	0.0684	0.0994
Dashuiyugou	DSY	31.74	1.2435	1.0573	0.0804	0.0704
Chenjiagou	CJG	28.75	1.4232	1.2048	0.0643	0.0544
Longwangmiao	LWM	33.62	1.2577	1.0735	0.0422	0.0722
Yinmagou	YMG	23.52	1.4565	1.2381	0.0971	0.061
Hualingou	HLG	23.34	1.1842	1.1088	0.0387	0.0603
Huojigou	HJG	30.96	1.0761	1.0182	0.0541	0.0244
Shijiagoucun	SJG	20.73	1.1253	1.0611	0.0412	0.0431
Caotangou	CTG	26.42	1.1088	1.0826	0.0315	0.0533
Liujiaping	LJP	25.93	1.2165	1.0802	0.0573	0.0961
Average		27.33	1.3645	1.1630	0.0621	0.0637
Total		56.02	1.7801	1.3732	0.1434	0.2362

**Note**: A_o_, observed number of alleles per locus; A_e_, effective number of alleles per locus; He, Nei’s gene diversity; H_o_, Shannon’s information index; PPB, percentage of polymorphic bands.

### Genetic structure and differentiation of the populations

#### UPGMA cluster analysis

**Figure 2:**
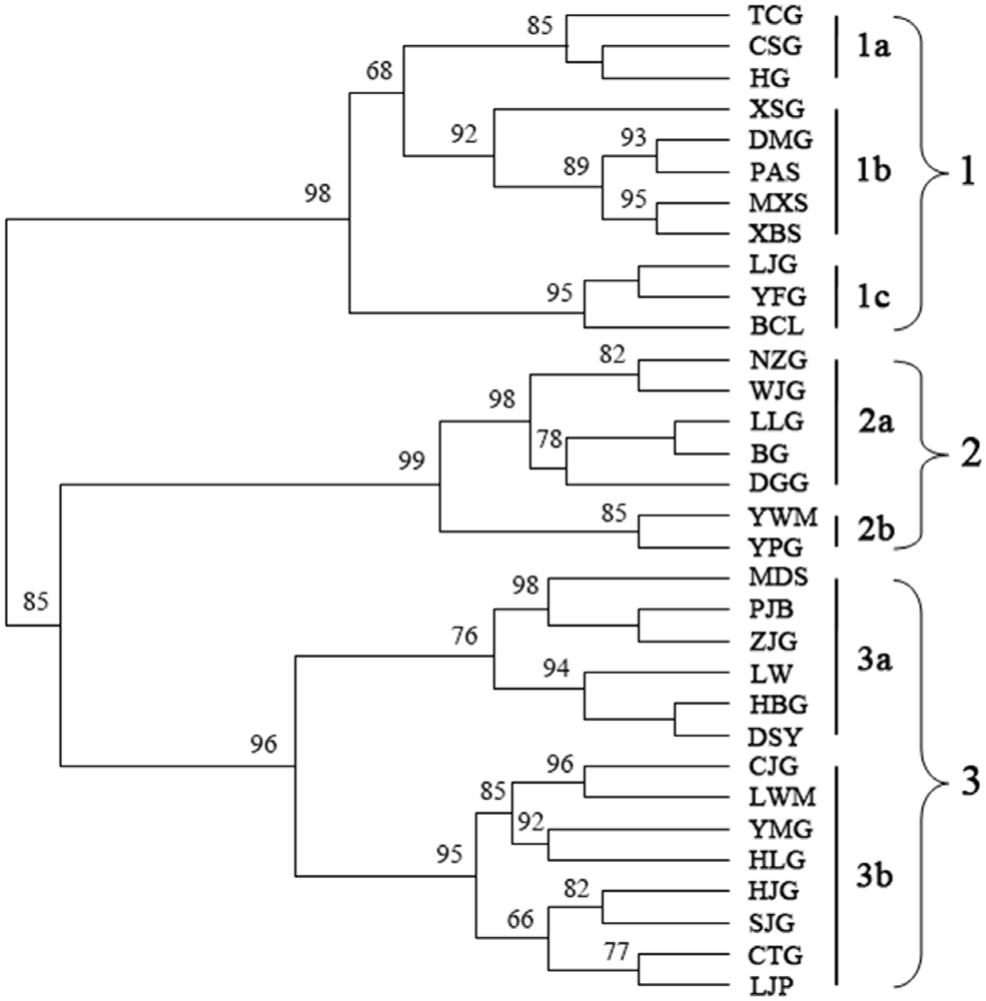
UPGMA clustering of *S. hexandrum* populations in Qinling Mts.

UPGMA clustering analysis defined three major groups among 32 populations (Figure 2). Group 1 included 11 populations, which were further divided into three subgroups. Populations TCG, CSG and HG were in subgroup 1a; populations XSG, DMG, PAS, MXS and XBS were in subgroup 1b; and populations LJG, YFG, and BCL were in subgroup 1c. Populations NZG, WJG, LLG and BG (Zhouzhi County), population DGG (Huxian County), population YWM (Taibai County) and population YPG (Huxian County) were grouped in cluster 2. Group 3 contained 14 populations, which were sampled from two adjacent cities, Baoji and Tianshui, and were subdivided into two clusters. Populations MDS, PJB, ZJG, LW, and HBG (Baoji) and DSY (Tianshui) were in subgroup 3a. Populations LWM, HLG, HJG, SJG, CTG, and LJP (Tianshui) and CJG, YMG (Baoji) were in subgroup 3b. Seven populations including DSY, LWM, HLG, HJG, SJG, CTG, and LJP in Group 3 were from Gansu Province. The other populations clustered in Groups 1, 2, and 3 were from Shaanxi Province. The *S. hexandrum* population was not clustered on the UPGMA tree according to geographic distance, which may indicate no obvious correlation between geographic distribution and genetic distance.

#### Principle coordinate analysis

**Figure 3:**
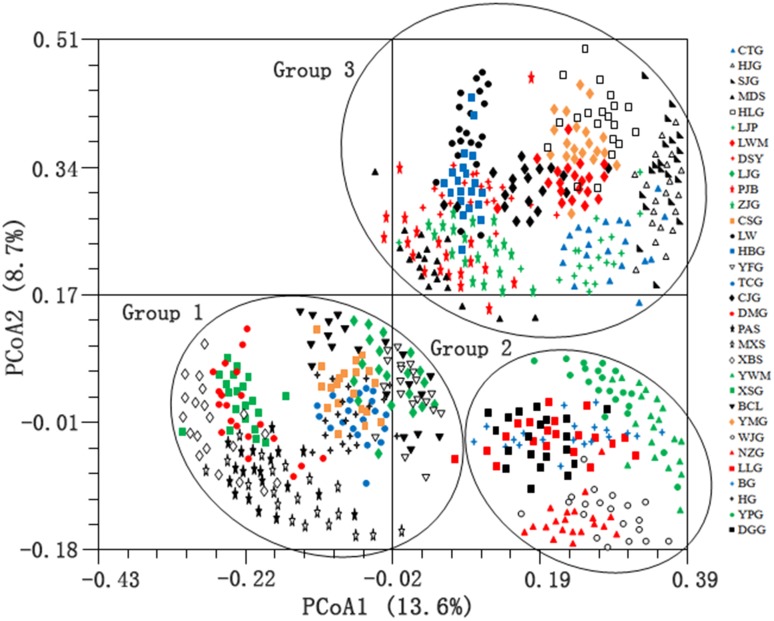
Distribution of individuals of the 32 *S. hexandrum* populations from Qinling Mts., according to the first (PCo1) and second (PCo2) principal coordinates. PCo1 and PCo2 account for 13.6 and 8.7% of the total variation, respectively.

PCoA was used for ordination and exploration of the similarity between populations. All samples were clearly separated into three major groups on the first (PCo1) and second (PCo2) principal coordinates (Figure 3). PCo1 explained 13.6% of the total variance, and PCo2 explained 8.7% of the total variance. Groups 1 and 2 are clustered within each other’s vicinity even though clearly separated, indicating higher similarity between these two groups. The clustering of the populations was in agreement with the UPGMA dendrogram.

#### Mantel test for IBD

**Figure 4:**
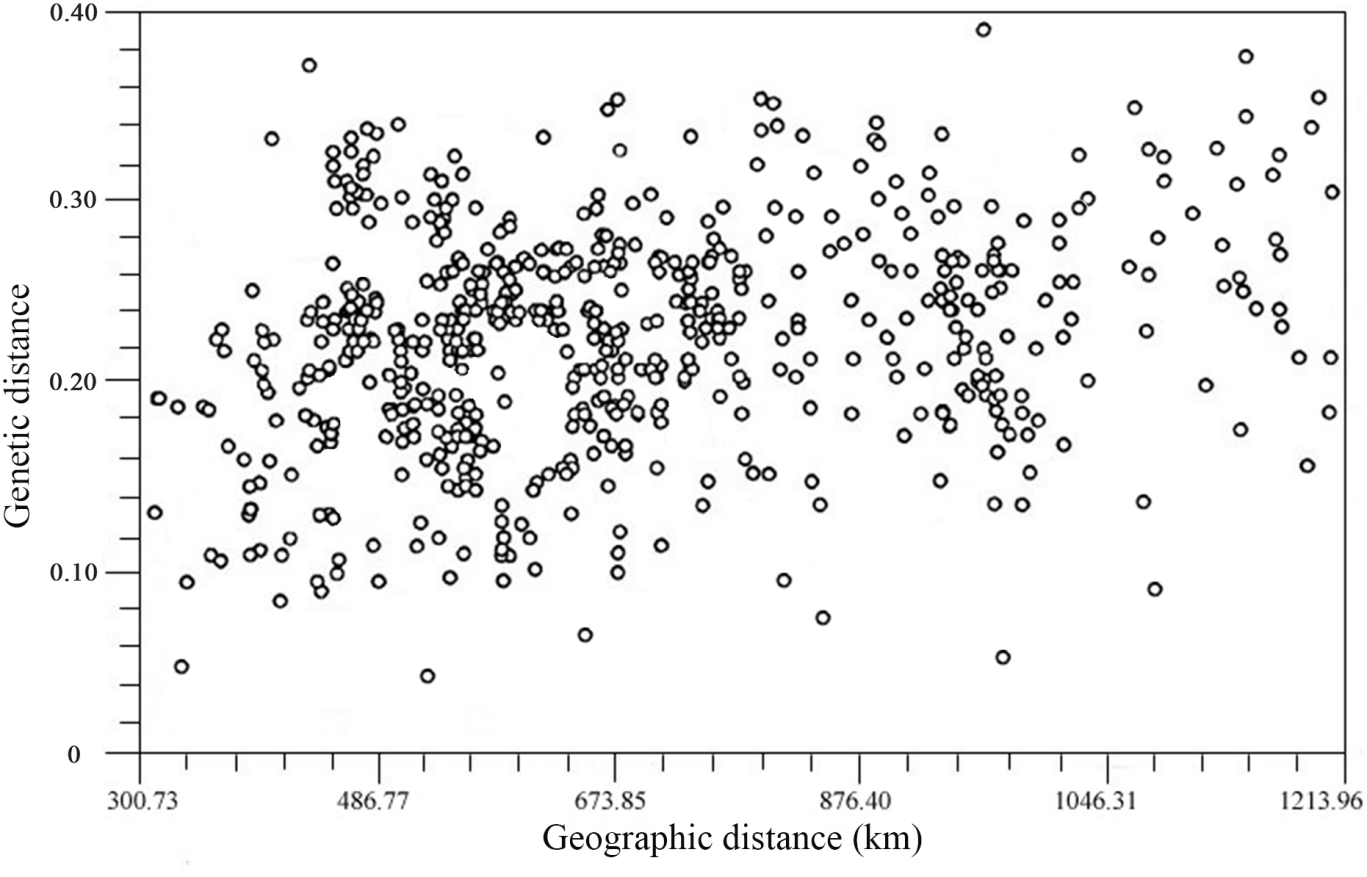
Mantel regression of the pairwise relationship between genetic and geographical distances for *S. hexandrum* populations.

A Mantel test for IBD was performed to assess the correlation between the genetic distance matrix and the corresponding geographic distance matrix of the wild *S. hexandrum* populations. The correlation coefficient *r* of genetic distance and geographic distance was 0.212 (*P* = 0.287), and the correlation analysis diagram (Figure 4) was comprised of many disordered and scattered points, indicating that the IBD of the wild *S. hexandrum* populations in Qinling Mts. was not significant at the level of 0.05. The Mantel test did not indicate correlation between genetic distance and geographical provenance.

#### Bayesian clustering

**Figure 5:**
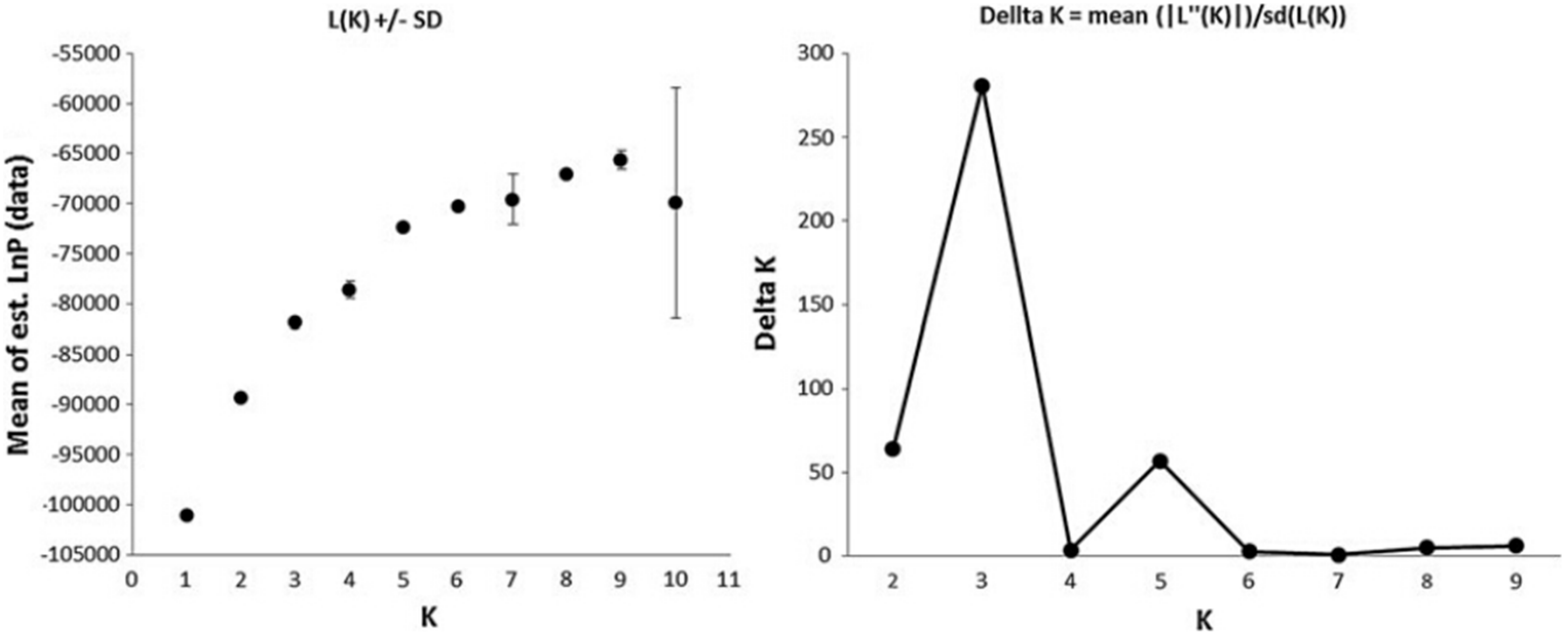
The probable K value estimated by likelihood of the probability of data L(K) and ad hoc quantity ΔK.

**Figure 6:**
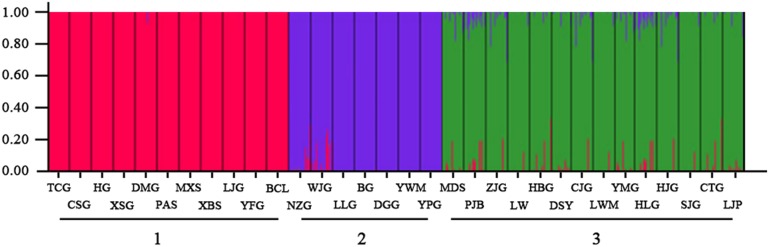
Bayesian clustering for infering population structure of *S. hexandrum* populations from Qinling Mts.

The genetic structure of the *S. hexandrum* samples were further analyzed using Bayesian clustering algorithm in the STRUCTURE software. The ΔK method indicated that the most likely K value was 3 (Figure 5). Sharp divisions were observed for the three clusters (Figure 6). The assignments of the populations to Groups 1, 2, and 3 were stable and consistent with UPGMA and PCoA clustering.

#### Analysis of molecular variance

The pairwise GD values (Table 4) were small and ranged from 0.0467 (XSG and DMG) to 0.3997 (BG and LW), indicating low differentiation within populations. Low population differentiation indicated that gene flow within each clustered group may be high or that isolation time was recent.

**Table 4:** Pairwise genetic distance estimates from 241 ISSR markers in *S. hexandrum*.

Population	TCG	CSG	HG	XSG	DMG	PAS	MXS	XBS	LJG	YFG	BCL	NZG	WJG	LLG	BG	DGG	YWM	YPG	MDS	PJB	ZJG	LW	HBG	DSY	CJG	LWM	YMG	HLG	HJG	SJG	CTG	LJP
TCG	***																															
CSG	0.1362	***																														
HG	0.0712	0.1379	***																													
XSG	0.1756	0.1444	0.1064	***																												
DMG	0.1833	0.1433	0.1343	0.0467	***																											
PAS	0.1654	0.1373	0.1312	0.0513	0.1514	***																										
MXS	0.1532	0.1920	0.1255	0.0568	0.2222	0.0634	***																									
XBS	0.1743	0.1636	0.1168	0.0764	0.3867	0.0754	0.0822	***																								
LJG	0.1503	0.1520	0.1205	0.0608	0.1290	0.0879	0.1075	0.0732	***																							
YFG	0.1853	0.1585	0.1484	0.0654	0.1640	0.0819	0.1015	0.0797	0.1755	***																						
BCL	0.1897	0.1574	0.1453	0.0709	0.1684	0.1575	0.1262	0.0786	0.1799	0.2085	***																					
NZG	0.1974	0.1514	0.1396	0.0905	0.1461	0.1786	0.1278	0.3726	0.1876	0.1907	0.1586	***																				
WJG	0.2095	0.2061	0.1309	0.0680	0.3582	0.1546	0.1093	0.1273	0.1697	0.1790	0.1555	0.3824	***																			
LLG	0.1673	0.1777	0.2213	0.1457	0.3891	0.1896	0.1158	0.0989	0.1830	0.1994	0.1498	0.2020	0.1616	***																		
BG	0.2448	0.1592	0.2445	0.1568	0.3483	0.1940	0.1147	0.3804	0.1885	0.1907	0.1411	0.3772	0.2163	0.2304	***																	
DGG	0.2727	0.1657	0.2449	0.1368	0.1980	0.2017	0.1087	0.0869	0.2081	0.1829	0.1422	0.1818	0.1879	0.2020	0.2035	***																
YWM	0.2696	0.1646	0.2185	0.1356	0.2179	0.2138	0.1334	0.0858	0.1815	0.1772	0.1384	0.1873	0.1763	0.1835	0.1948	0.2076	***															
YPG	0.2639	0.1586	0.3831	0.1431	0.1844	0.1716	0.1350	0.0798	0.1861	0.1685	0.2015	0.2069	0.1828	0.1900	0.3893	0.2272	0.2040	***														
MDS	0.1552	0.2133	0.3086	0.0830	0.2136	0.2014	0.1142	0.1345	0.1624	0.1612	0.2136	0.1784	0.1817	0.1889	0.2172	0.2047	0.2251	0.2114	***													
PJB	0.2520	0.1849	0.3223	0.1026	0.2202	0.1462	0.1207	0.1061	0.1453	0.1891	0.1696	0.3726	0.2137	0.1829	0.2141	0.2093	0.1991	0.2318	0.2197	***												
ZJG	0.1799	0.1641	0.3050	0.0741	0.1530	0.1127	0.0896	0.3760	0.1674	0.1860	0.1694	0.0781	0.1853	0.2376	0.2084	0.1729	0.2341	0.2231	0.3793	0.1879	***											
LW	0.2768	0.1706	0.3384	0.0787	0.1413	0.0843	0.1136	0.0971	0.1927	0.1803	0.1524	0.0977	0.1405	0.2092	0.3997	0.2079	0.2385	0.2474	0.2108	0.1822	0.2160	***										
HBG	0.1778	0.1695	0.1430	0.0842	0.1617	0.0753	0.1683	0.3744	0.1667	0.2150	0.1747	0.0729	0.2006	0.1884	0.1905	0.2123	0.2162	0.1946	0.2154	0.1735	0.2206	0.2416	***									
DSY	0.1574	0.1635	0.3868	0.1394	0.1530	0.0818	0.2399	0.0540	0.2017	0.1510	0.1711	0.0775	0.1307	0.1949	0.2184	0.2200	0.2283	0.2011	0.3709	0.1772	0.2261	0.2208	0.2214	***								
CJG	0.1925	0.2182	0.1617	0.1110	0.3773	0.0807	0.1154	0.3891	0.2061	0.1556	0.1624	0.1916	0.1769	0.1938	0.2153	0.2021	0.2161	0.2000	0.2405	0.2051	0.2457	0.2273	0.2461	0.0884	***							
LWM	0.1963	0.1898	0.1586	0.0865	0.1169	0.0747	0.1219	0.0929	0.1838	0.1611	0.1569	0.2112	0.1676	0.1878	0.2096	0.1899	0.2372	0.1940	0.2139	0.2020	0.2087	0.2262	0.2177	0.0624	0.0477	***						
YMG	0.2042	0.1653	0.1529	0.1514	0.3719	0.1294	0.0908	0.1008	0.1959	0.1807	0.1848	0.1836	0.1757	0.2425	0.2009	0.2110	0.2145	0.2487	0.3815	0.1963	0.2152	0.2202	0.1631	0.0974	0.0756	0.1038	***					
HLG	0.1864	0.1718	0.1442	0.1410	0.1563	0.1010	0.1148	0.0930	0.1837	0.1651	0.1817	0.1882	0.1880	0.2141	0.1936	0.1870	0.1941	0.2203	0.2240	0.1876	0.2141	0.2749	0.1910	0.1018	0.0725	0.0772	0.1090	***				
HJG	0.1747	0.1707	0.1369	0.1481	0.1640	0.0825	0.1395	0.0919	0.2048	0.1697	0.1760	0.3937	0.1935	0.1896	0.2215	0.2220	0.2292	0.2148	0.2436	0.1844	0.2081	0.2465	0.0774	0.0795	0.0668	0.0818	0.0874	0.1415	***			
SJG	0.0991	0.1647	0.3648	0.1470	0.1761	0.0890	0.1111	0.0859	0.1821	0.1752	0.1673	0.2133	0.2131	0.1961	0.2184	0.2264	0.2330	0.2344	0.2159	0.2123	0.2628	0.2220	0.0717	0.0916	0.0581	0.0873	0.0939	0.1131	0.0940	***		
CTG	0.1864	0.1894	0.1617	0.1722	0.1339	0.1366	0.1021	0.1106	0.1617	0.1948	0.1581	0.1622	0.1975	0.1950	0.2127	0.2341	0.2409	0.2096	0.3624	0.2092	0.2344	0.2285	0.0839	0.0794	0.0526	0.1069	0.0928	0.0886	0.0880	0.0853	***	
LJP	0.2107	0.1610	0.1560	0.1686	0.3655	0.1082	0.1086	0.3822	0.1968	0.1723	0.1860	0.1687	0.2021	0.1890	0.3834	0.2462	0.2231	0.2142	0.2213	0.2153	0.2700	0.2274	0.0894	0.1005	0.0805	0.0793	0.0868	0.0951	0.0847	0.0918	0.0907	***

AMOVA (Table 5) was also performed for population differentiation to further evaluate genetic structure. Highly significant (*P*<0.000 2) genetic variance was expectedly observed among the populations and explained 63.27% of the total variance, supporting the results from the hierarchical and Bayesian clustering. Only 36.73% of the total genetic variance occurred within populations, indicating higher genetic differentiation between the populations than within each population and the emergence of genetic differentiation among populations. The G_st_ value (0.5823) (Table 2) showed there were more variation among populations than that within populations, confirming the AMOVA results.

**Table 5:** Analysis of molecular variance (AMOVA) for 640 individuals in 32 populations of *S. hexandrum* using 11 selected ISSR primers.

Source of variation	df	Sum of squares	Mean squares	Variance component	Total variance (%)	*P*-value
Among population	31	4,028.326	129.946	3.9128	63.27	<0.000 2
Within population	608	950.912	1.564	1.564	36.73	<0.000 2

## Discussion

### Genetic diversity of *S. hexandrum* in Qinling Mts

The ISSR markers developed in this study effectively revealed low genetic diversity within the *S. hexandrum* populations sampled in Qinling Mts. Populations are isolated given the population differentiation and clear clustering. The comparison of the average genetic diversity to that of *S. hexandrum* from Northwestern Himalayan region [34], [36], [38] and other Berberidaceae species [58], [59] based on the ISSR approach showed that *S. hexandrum* populations of Qinling Mts. have low genetic diversity (average H_e_ = 0.0621). The *S. hexandrum* in the Northwestern Himalayan region [34], [36], [38] showed high genetic variation (H_e_ = 0.2944, 0.092, respectively). *S. hexandrum* is native to the Himalayan region, growing in valleys with secondary vegetation, or under shrubs or around trees [2]. Their habitat is significantly different from that of Qinling Mts. *S. hexandrum* reproduces through vegetative reproduction and seeds. Insects and birds are limited in the high altitude regions, implying that *S. hexandrum* pollination is easier than in the Qingling Mts. The sizes of wild populations of *S. hexandrum* are very small and declines each year in Qinling Mts. because of habitat fragmentation and deterioration caused by human disturbance (overcollection due to economic interests). The rapid decrease in individuals in wild populations may also cause loss of the genetic diversity of this endangered species. Another Berberidaceae species, *Dysosma versipellis*, is an endangered species endemic to China and has been listed as a key protected wild plant in China due to habitat fragmentation or destruction. Qiu et al. found this species had high level of genetic diversity in China (H_e_ = 0.378) [58]. *Dysosma pleiantha*, a threatened medicinal plant species distributed in southeastern China, sexually and asexually reproduces. High H_e_ (0.364) was observed in this species [59]. The relatively high level of genetic variation observed within two species suggested that the balance between vegetative reproduction and sexual reproduction was more in favor of sexual reproduction in the populations *D. versipellis* and *D. pleiantha* than in the *S. hexandrum* populations. The H_e_ found in this study was 0.1434 at the species level, lower than those of some strictly self-pollinating soybean species (H_e_ = 0.1714) [60] and the self-pollinating *Oryza granulata* (H_e_ = 0.210) [61], which also indicated that potential selfing system in these populations reduced genetic diversity of *S. hexandrum* populations. Historical events are also responsible for the variation in genetic diversity [62].

Genetic diversity is affected by multiple factors, such as geographical distribution, mating system, life form, pollen and seed dispersal [63], [64]. Low genetic variation within populations could be attributed to seed dispersal and the predominant clonal reproduction in *S. hexandrum* in this high mountainous area. However, the genetic diversity of *S. hexandrum* is not much lower than other endangered species analyzed using ISSR markers, such as *Leontice microrhyncha* (Berberidaceae) (H_e_ = 0.021), which is a polycarpic perennial herb found in deciduous or coniferous forests in Korea and Northeast China [65]. For species *L. microrhyncha,* each pollinated flower produces an 8 mm berry and seed dispersal is restricted due to its heavy berry [66]. The genetic diversity of *S. hexandrum* was also higher than those of two other species, the endangered *Pinus squamata* (H_e_ = 0.020) [67] and the first-degree endangered species *Manglietia decidua* (H_e_ = 0.0637) [68]. *P. squamata* and *M. decidua* are extremely rare and endangered tree species in China. The extremely low genetic diversity of this two species could have resulted from the severe bottleneck effect during their evolutionary process. The gene drift and inbreeding may further decrease their genetic diversity in the shrinking populations. The weak competitive ability against broad - leaved trees and human activities may also accelerated the decrease of genetic variation.

*S. hexandrum* is reasonably long lived because its rhizomes easily reproduce, which could slow down the loss of genetic diversity. Pollen dispersal is generally restricted to a small region due to the large pollen size, which limits gene flow to increase or maintain genetic diversity. *S. hexandrum* is native to the Himalayan region, including China, India, Nepal, and Myanmar. No other genetic diversity studies of *S. hexandrum* exist in other locations. Further research should include more populations in other regions of China and countries.

### High genetic differentiation and distinct genetic structure

High level of genetic differentiation and clear population structure was detected in this study. K = 3 in the Bayesian clustering as a meaningful value because group 1, 2 and 3 could be divided by detecting the within-group substructure. This result is also supported by distance-based clustering and PCoA. Groups 1 and 2 are genetically close but significantly different. Estimation of the number of clusters K should be treated with care because it is computationally difficult to obtain accurate estimates and the method merely provides an ad hoc approximation [52].

All sampled individuals were strongly assigned to their original populations, and all data strongly support the conclusion that the 32 *S. hexandrum* populations distributed in the Qinling Mts. are clustered into three major groups. These methods consistently showed that high genetic differentiation existed among *S. hexandrum* populations, which is consistent with genetic variation studies in certain selfing species [69]. This would mean that *S. hexandrum* should be a selfing species or a selfing predominant species, which is consistent with previous studies on *S. hexandrum* by Ma et al. [70]. Aside from the breeding system, the high genetic differentiation across populations may also be caused by genetic drift [71]. Wright [72] noted that genetic drift would lead a small population to emerge with a distinct genetic differentiation when the N_m_ value is lower than 1.0. The N_m_ of *S. hexandrum* (0.3587) determined using the POPGENE software was lower than 1.0 in the present study, which suggested that some genetic drift may have emerged among the populations of this species. The distribution of *S. hexandrum* populations obviously tend to fragment based on the field investigation, which is consistent with the possibility of genetic drift.

Migration of plant populations can occur through dispersal of pollen and seed [73]. But a number of factors such as fragmented geographical distribution, lack of pollinators or seed dispersers can be a barrier to gene flow between populations [74], [75]. Limited gene flow among *S. hexandrum* populations may be related to inbreeding of the species and limited seed propagation distance. Some studies have found that seed dispersal is the primary factor influencing variation of gene flow and population structure [76]. Heavy mature berries of *S. hexandrum* usually drop to the ground because of rain or wind, settling some seeds in the soil, whereas others are dispersed by cattle, birds, or humans. Therefore, the short distance of seed dispersal of *S. hexandrum* probably resulted in limited gene flow among populations. Mountain ranges and rivers are possible barriers to either dispersal of pollen or rhizomes of *S. hexandrum*, reproductively isolating the populations. The restriction of gene flow associated with geographical distance is consistent with the results of previous studies on this species [34], [35], [36].

### Implications for conservation

*S. hexandrum* is a rare and threatened species [4] in China. Assessment of genetic diversity is important for designing conservation strategies for threatened and endangered species [77], [78]. The results of this study showed that there was low genetic diversity among *S. hexandrum* populations and genetic differentiation among populations was higher than within populations. Genetic diversity loss has deleterious effects on species fitness and threatens the population survival and could be the key reason that explains the endangerment of *S. hexandrum* in Qinling Mts. [79], [80]. The estimation of the genetic diversity and population genetic structure could provide bases for *S. hexandrum* conservation and its reasonable utilization. The results will help determine what to conserve and where and how to conserve this species.

The field survey showed that the habitats of some populations have been destroyed by human disturbance for great medical value. Damage to natural habitats would led to a decrease in population sizes and probably a subsequent increase in inbreeding, decreasing its genetic diversity. In situ conservation effectively and sustainably prevents this problem. The establishment of *S. hexandrum* reserves should be the primary method because the Qinling Mts. are situated in state forest conservation areas, where cutting and hunting are restricted. The management for the conservation of genetic variability in this species should aim to preserve not only large populations but also as many of the small populations outside nature reserves as possible. Reduced levels of genetic variation, especially in the smaller populations, will affect the species’ ability to adapt to changes in its habitat [81]. Positive correlations between population size, expected heterozygosity, and plant fitness were found in *Gentiana pneumonanthe* [82] and *Arnica Montana* [81]. Thus, policy plans should also be developed to stimulate seedling recruitment in the small populations (e.g., PAS and MXS). It may be dangerous to mix highly divergent populations because it could cause loss of adaptive diversity [45]. Therefore, it is necessary to improve gene flow among populations within each group through some artificial means, such as transplanting individuals (by seed, rhizomes from one population to another). Furthermore, to avoid human overcollection, greater awareness for *S. hexandrum* protections must be emphasized, and related forest departments should be encouraged to undertake conservation through an integrated conservation strategy based on demographic, ecological, and genetic aspects.

As a supplement to in situ conservation, ex situ conservation would also be feasible as underlined by other studies on endangered species [83], [84], [85]. Populations may be partially preserved through seed banks or in vitro germplasm collections. *S. hexandrum* has favorable sexual reproduction. Each plant produces approximately 60 seeds, with a maximum of approximately 180 seeds [70]. Seed collection is easier for *S. hexandrum* than other endangered species. Thus, a strategy involving extensive collection to ensure full sampling of genetic diversity, subsequent cultivation in a garden at least 1000 m above sea level [7], and reintroduction into their original wild habitats seems feasible, although *S. hexandrum* mainly grows wild on high altitude mountain ranges. For *S. hexandrum* populations in Qinling Mts., there are some preserved forest farms which could be used for relocation. However, ex situ conservation has many drawbacks because it is impossible to recreate the habitat as a whole. The new environment may have important ecological differences compared with the original habitat, and the approach is technically challenging and is often expensive. Therefore, ex situ conservation is recommended only to supplement in situ conservation or as a last resort. In vitro techniques are also proven to be an effective alternative means of propagation that facilitates the recovery of the rare and endangered *S. hexandrum* [33]. At present, an effective protocol of in vitro propagation, involving multiple shoot formation from zygotic embryos and subsequent rooting, could be available for *S. hexandrum*. In vitro propagation may well be used as a means to rescue zygotic embryos for this species. In vitro techniques induce variability, but plants raised from tissue cultures may be screened for useful somaclonal variants and exploited to obtain plants or cultures with high podophyllotoxin contents, which possibly reduces the pressure on natural *S. hexandrum* populations.

Most of the genetic diversity of the important medicinal and endangered species *S. hexandrum* in Qinling Mts. must be guaranteed with these combined and sustained efforts.

## Supporting Information

Text S1 **Description of the sampling procedures.**
(DOC)

Text S2 **Allele frequencies per locus.**
(DOC)
