# Supplementary material for: Genetic Diversity and Structure of Sinopodophyllum hexandrum (Royle) Ying in the Qinling Mountains, China
Source: PLoS One. 2014 Oct 15;9(10):e110500. doi: 10.1371/journal.pone.0110500 (PMC4198291; doi:10.1371/journal.pone.0110500)
Supplement: Text S1 — Description of the sampling procedures. (DOC) [file pone.0110500.s001.doc]

**Genetic Diversity and Structure of *Sinopodophyllum hexandrum* (Royle) Ying in the Qinling Mountains, China**

(Supporting Information Text S1)

**Wei Liu, Dongxue Yin, Jianjun Liu[[1]](#footnote-2)*, Na Li**

College of Forestry, Northwest A & F University, Yangling, China

**Collection permission of experiment materials**

*Sinopodophyllum hexandrum* in the present study is one of endangered species in China. Currently, with the enhanced awareness of medicinal value for peoples’ health and the discovery of better efficacy in clinic application, the availability of *S. hexandrum* resource has become increasingly limited due to both its intense collection from nature as the commercial source of podophyllotxion, habitat destruction and the lack of organized cultivation in China. *S. hexandrum* has been classified as an endangered species (grade 3) in 1987 by the Chinese Plant Red Book. This work was supported by the special scientific research project of national forestry public welfare profession of China (Grant No.: 200904004). Its aims were to optimized exploitation, utilization and protection of wild medicinal resources in Qinling mountains. *S. hexandrum* was considered as one of research objectives. Thus, there was no specific permissions to be required in this study. We just need to do like this to collect plant materials: Firstly, the authority of Northwest university of A & F issued a certificate of the investigation and research for each site. Secondly, the local corresponding management department checked and registrated the information, and then “Enter Proof” was issued. Thirdly, we entered into the production locations for sampling. The names of the authorities that issued the permits for each location were listed in Table S1.

**Table S1.** The name of the authority that issued the permit for each location.

| No. | Population | Code | Coordinates | Altitude  /m | Permit affiliation | Address |
| --- | --- | --- | --- | --- | --- | --- |
| 1 | Tancaogou | TCG | E105°48′N34°21′ | 1 899 | Maiji district forestry bureau | Fengxian, Baoji |
| 2 | Chunshugou | CSG | E106°53′N34°6′ | 1 728 | Fengxian forest bureau | Fengxian, Baoji |
| 3 | Hougou | HG | E107°1′N34°2′ | 1 512 | Fengxian forest bureau | Fengxian, Baoji |
| 4 | Xiaoshagou | XSG | E108°33′N33°55′ | 1 439 | Management bureau of Taibai Mt. | Taibai, Baoji |
| 5 | Doumugong | DMG | E107°27′N34°1′ | 2 332 | Management bureau of Taibai Mt. | Meixian, Baoji |
| 6 | Pinganssi | PAS | E107°43′N34°2′ | 2 748 | Management bureau of Taibai Mt. | Meixian, Baoji |
| 7 | Mingxingsi | MXS | E107°43′N34°1′ | 2 815 | Management bureau of Taibai Mt. | Meixian, Baoji |
| 8 | Xiabansi | XBS | E107°44′N34°0′ | 2 637 | Management bureau of Taibai Mt. | Meixian, Baoji |
| 9 | Laojungou | LJG | E107°47′N33°59′ | 3 483 | Liangdang forestry bureau | Liangdang, Baoji |
| 10 | Youfanggou | YFG | E106°33′N34°9′ | 1 902 | Fengxian forest bureau | Fengxian, Baoji |
| 11 | Baicaoling | BCL | E106°40′N34°12′ | 1 545 | Management bureau of Taibai Mt. | Taibai, Baoji |
| 12 | Nianzigou | NZG | E107°24′N34°9′ | 1 373 | Zhouzhi forestry bureau | Zhouzhi, Xi’an |
| 13 | Wenjiagou | WJG | E108°6′N33°56′ | 1 821 | Zhouzhi forestry bureau | Zhouzhi, Xi’an |
| 14 | Liulingou | LLG | E108°5′N33°59′ | 1 765 | Zhouzhi forestry bureau | Zhouzhi, Xi’an |
| 15 | Beigou | BG | E108°10′N33°52′ | 1 013 | Zhouzhi forestry bureau | Zhouzhi, Xi’an |
| 16 | Dagangou | DGG | E108°16′N33°51′ | 1 579 | Huxian forestry bureau | Huxian, Xi’an |
| 17 | Yaowangmiao | YWM | E108°47′N33°55′ | 1 335 | Management bureau of Taibai Mt. | Taibai, Baoji |
| 18 | Yingpangoukou | YPG | E107°31′N33°53′ | 1 779 | Huxian forestry bureau | Huxian, Xi’an |
| 19 | Maiduoshigou | MDS | E108°34′N33°47′ | 1 487 | Liangdang, forestry bureau | Liangdang, Baoji |
| 20 | Panjiaba | PJB | E106°7′N34°10′ | 1 520 | Liangdang, forestry bureau | Liangdang, Baoji |
| 21 | Zhangjiagou | ZJG | E106°20′N34°10′ | 1 976 | Fengxian forest bureau | Fengxian, Baoji |
| 22 | Longwangmiao | LW | E107°5′N34°1′ | 1 469 | Fengxian forest bureau | Fengxian, Baoji |
| 23 | Huangbaigou | HBG | E106°57′N33°55′ | 1 475 | Fengxian forest bureau | Fengxian, Baoji |
| 24 | Dashuiyugou | DSY | E106°59′N34°6′ | 1 745 | Maiji district forestry bureau | Maiji, Tianshui |
| 25 | Chenjiagou | CJG | E106°30′N34°18′ | 1 878 | Fengxian forest bureau | Fengxian, Baoji |
| 26 | Longwangmiao | LWM | E106°46′N34°13′ | 1 515 | Maiji district forestry bureau | Maiji, Tianshui |
| 27 | Yinmagou | YMG | E106°23′N34°15′ | 1 736 | Management bureau of Taibai Mt. | Taibai, Baoji |
| 28 | Hualingou | HLG | E107°11′N33°58′ | 2 252 | Maiji district forestry bureau | Maiji, Tianshui |
| 29 | Huojigou | HJG | E106°17′N34°18′ | 1 793 | Maiji district forestry bureau | Maiji, Tianshui |
| 30 | Shijiagoucun | SJG | E106°2′N34°19′ | 1 718 | Maiji district forestry bureau | Maiji, Tianshui |
| 31 | Caotangou | CTG | E105°56′N34°25′ | 1 366 | Maiji district forestry bureau | Maiji, Tianshui |
| 32 | Liujiaping | LJP | E105°44′N34°30′ | 1 690 | Maiji district forestry bureau | Maiji, Tianshui |

1. ^^ Correspondence author

   E-mail: [ljj@nwsuaf.edu.cn](mailto:ljj@nwsuaf.edu.cn) [↑](#footnote-ref-2)
