# Supplementary material for: Genetic Diversity and Structure of Sinopodophyllum hexandrum (Royle) Ying in the Qinling Mountains, China
Source: PLoS One. 2014 Oct 15;9(10):e110500. doi: 10.1371/journal.pone.0110500 (PMC4198291; doi:10.1371/journal.pone.0110500)
Supplement: Text S2 — Allele frequencies per locus. (DOC) [file pone.0110500.s002.doc]

**Genetic Diversity and Structure of *Sinopodophyllum hexandrum* (Royle) Ying in the Qinling Mountains, China**

(Supporting Information Text S2)

**Wei Liu, Dongxue Yin, Jianjun Liu[[1]](#footnote-2)*, Na Li**

College of Forestry, Northwest A & F University, Yangling, China

**Table S2.** Allele frequencies per locus.

| Allele\Locus | 1 | 2 | 3 | 4 | 5 | 6 | 7 | 8 | 9 | 10 | 11 | 12 | 13 | 14 | 15 | 16 | 17 | 18 | 19 | 20 | 21 | 22 | 23 | 24 | 25 | 26 | 27 | 28 | 29 | 30 | 31 | 32 |
| --- | --- | --- | --- | --- | --- | --- | --- | --- | --- | --- | --- | --- | --- | --- | --- | --- | --- | --- | --- | --- | --- | --- | --- | --- | --- | --- | --- | --- | --- | --- | --- | --- |
| Allele 0 | 0.8011 | 0.4861 | 0.8746 | 0.8021 | 0.513 | 0.0177 | 0.5157 | 0.3256 | 0.4107 | 0.8717 | 0.2653 | 0.8151 | 0.4101 | 0.2765 | 0.6831 | 0.2507 | 0.7006 | 0.3856 | 0.7741 | 0.7016 | 0.4125 | 0.7028 | 0.4152 | 0.2251 | 0.3102 | 0.7712 | 0.1648 | 0.7146 | 0.6538 | 0.176 | 0.5826 | 0.1502 |
| Allele 1 | 0.1989 | 0.5139 | 0.1254 | 0.1979 | 0.487 | 0.9823 | 0.4843 | 0.6744 | 0.5893 | 0.1283 | 0.7347 | 0.1849 | 0.5899 | 0.7235 | 0.3169 | 0.7493 | 0.2994 | 0.6144 | 0.2259 | 0.2984 | 0.5875 | 0.2972 | 0.5848 | 0.7749 | 0.6898 | 0.2288 | 0.8352 | 0.2854 | 0.3462 | 0.824 | 0.4174 | 0.8498 |
| Allele\Locus | 33 | 34 | 35 | 36 | 37 | 38 | 39 | 40 | 41 | 42 | 43 | 44 | 45 | 46 | 47 | 48 | 49 | 50 | 51 | 52 | 53 | 54 | 55 | 56 | 57 | 58 | 59 | 60 | 61 | 62 | 63 | 64 |
| Allele 0 | 0.7911 | 0.4761 | 0.8646 | 0.7921 | 0.501 | 0.0077 | 0.5057 | 0.3134 | 0.4007 | 0.8617 | 0.2553 | 0.8051 | 0.6635 | 0.2665 | 0.6729 | 0.2407 | 0.6512 | 0.7058 | 0.5793 | 0.3074 | 0.3096 | 0.3868 | 0.0692 | 0.6164 | 0.4769 | 0.4911 | 0.7945 | 0.7812 | 0.4411 | 0.7406 | 0.7582 | 0.7648 |
| Allele 1 | 0.2089 | 0.5239 | 0.1354 | 0.2079 | 0.499 | 0.9923 | 0.4943 | 0.6866 | 0.5993 | 0.1383 | 0.7447 | 0.1949 | 0.3365 | 0.7335 | 0.3271 | 0.7593 | 0.3488 | 0.2942 | 0.4207 | 0.6926 | 0.6904 | 0.6132 | 0.9308 | 0.3836 | 0.5231 | 0.5089 | 0.2055 | 0.2188 | 0.5589 | 0.2594 | 0.2418 | 0.2352 |
| Allele\Locus | 65 | 66 | 67 | 68 | 69 | 70 | 71 | 72 | 73 | 74 | 75 | 76 | 77 | 78 | 79 | 80 | 81 | 82 | 83 | 84 | 85 | 86 | 87 | 88 | 89 | 90 | 91 | 92 | 93 | 94 | 95 | 96 |
| Allele 0 | 0.5511 | 0.6037 | 0.7272 | 0.2095 | 0.2073 | 0.2867 | 0.309 | 0.6233 | 0.3768 | 0.4192 | 0.6944 | 0.6811 | 0.4215 | 0.6427 | 0.6576 | 0.6647 | 0.6989 | 0.7535 | 0.3116 | 0.3551 | 0.3573 | 0.4345 | 0.1169 | 0.2187 | 0.5246 | 0.5021 | 0.8422 | 0.8289 | 0.6067 | 0.7836 | 0.8059 | 0.8125 |
| Allele 1 | 0.4489 | 0.3963 | 0.2728 | 0.7905 | 0.7927 | 0.7133 | 0.691 | 0.3767 | 0.6232 | 0.5808 | 0.3056 | 0.3189 | 0.5785 | 0.3573 | 0.3424 | 0.3353 | 0.3011 | 0.2465 | 0.6884 | 0.6449 | 0.6427 | 0.5655 | 0.8831 | 0.7813 | 0.4754 | 0.4979 | 0.1578 | 0.1711 | 0.3933 | 0.2164 | 0.1941 | 0.1875 |
| Allele \Locus | 97 | 98 | 99 | 100 | 101 | 102 | 103 | 104 | 105 | 106 | 107 | 108 | 109 | 110 | 111 | 112 | 113 | 114 | 115 | 116 | 117 | 118 | 119 | 120 | 121 | 122 | 123 | 124 | 125 | 126 | 127 | 128 |
| Allele 0 | 0.504 | 0.3569 | 0.2562 | 0.7968 | 0.089 | 0.6048 | 0.7417 | 0.8549 | 0.9309 | 0.816 | 0.8577 | 0.3807 | 0.841 | 0.9944 | 0.4206 | 0.5424 | 0.9012 | 0.5862 | 0.9747 | 0.9022 | 0.6131 | 0.1178 | 0.6158 | 0.4257 | 0.5108 | 0.9604 | 0.3654 | 0.9152 | 0 | 0.3744 | 0.7832 | 0.3508 |
| Allele 1 | 0.496 | 0.6431 | 0.7438 | 0.2032 | 0.911 | 0.3952 | 0.2583 | 0.1451 | 0.0691 | 0.184 | 0.1423 | 0.6193 | 0.159 | 0.0056 | 0.5794 | 0.4576 | 0.0988 | 0.4138 | 0.0253 | 0.0978 | 0.3869 | 0.8822 | 0.3842 | 0.5743 | 0.4892 | 0.0396 | 0.6346 | 0.0848 | 1 | 0.6256 | 0.2168 | 0.6492 |
| Allele \Locus | 129 | 130 | 131 | 132 | 133 | 134 | 135 | 136 | 137 | 138 | 139 | 140 | 141 | 142 | 143 | 144 | 145 | 146 | 147 | 148 | 149 | 150 | 151 | 152 | 153 | 154 | 155 | 156 | 157 | 158 | 159 | 160 |
| Allele 0 | 0.8793 | 0.9151 | 0.5541 | 0.0932 | 0.654 | 0.9495 | 0.9266 | 0.3822 | 0.547 | 0.8181 | 0.7625 | 0.6327 | 0.5549 | 0.3588 | 0.6422 | 0.4783 | 0.8375 | 0.4895 | 0.6024 | 0.624 | 0.8452 | 0.7822 | 0.9609 | 0.9022 | 0.0442 | 0.9918 | 4549 | 0.0469 | 0.9159 | 0.5564 | 0.6955 | 0 |
| Allele 1 | 0.1207 | 0.0849 | 0.4459 | 0.9068 | 0.346 | 0.0505 | 0.0734 | 0.6178 | 0.453 | 0.1819 | 0.2375 | 0.3673 | 0.4451 | 0.6412 | 0.3578 | 0.5217 | 0.1625 | 0.5105 | 0.3976 | 0.376 | 0.1548 | 0.2178 | 0.0391 | 0.0978 | 0.9558 | 0.0082 | -4548 | 0.9531 | 0.0841 | 0.4436 | 0.3045 | 1 |
| Allele \Locus | 161 | 162 | 163 | 164 | 165 | 166 | 167 | 168 | 169 | 170 | 171 | 172 | 173 | 174 | 175 | 176 | 177 | 178 | 179 | 180 | 181 | 182 | 183 | 184 | 185 | 186 | 187 | 188 | 189 | 190 | 191 | 192 |
| Allele 0 | 0.9732 | 0.6047 | 0.3501 | 0.3811 | 0 | 0.6094 | 0 | 0.5903 | 0.9019 | 0 | 0.1838 | 0.9653 | 0.9653 | 0.5073 | 0.6408 | 0 | 0.914 | 0.3651 | 0.9583 | 0 | 0.4427 | 0.6927 | 0.6927 | 0.5688 | 0.6927 | 0 | 0.5034 | 0.4115 | 0.4997 | 0.7809 | 0 | 0.6927 |
| Allele 1 | 0.0268 | 0.3953 | 0.6499 | 0.6189 | 1 | 0.3906 | 1 | 0.4097 | 0.0981 | 1 | 0.8162 | 0.0347 | 0.0347 | 0.4927 | 0.3592 | 1 | 0.086 | 0.6349 | 0.0417 | 1 | 0.5573 | 0.3073 | 0.3073 | 0.4312 | 0.3073 | 1 | 0.4966 | 0.5885 | 0.5003 | 0.2191 | 1 | 0.3073 |
| Allele \Locus | 193 | 194 | 195 | 196 | 197 | 198 | 199 | 200 | 201 | 202 | 203 | 204 | 205 | 206 | 207 | 208 | 209 | 210 | 211 | 212 | 213 | 214 | 215 | 216 | 217 | 218 | 219 | 220 | 221 | 222 | 223 | 224 |
| Allele 0 | 0.0943 | 0.3522 | 0.3752 | 0.9792 | 0.016 | 0.1699 | 0.9469 | 0.6853 | 0.7756 | 0.9887 | 0.45 | 0.7829 | 0.6275 | 0.692 | 0.6366 | 0.9357 | 0.2636 | 0.9375 | 0.1484 | 0.9068 | 0.7062 | 0.142 | 0.9886 | 0.4965 | 0.7504 | 0.8871 | 0.9718 | 0.6901 | 0.9666 | 0.2361 | 0.1284 | 0.8436 |
| Allele 1 | 0.9057 | 0.6478 | 0.6248 | 0.0208 | 0.984 | 0.8301 | 0.0531 | 0.3147 | 0.2244 | 0.0113 | 0.55 | 0.2171 | 0.3725 | 0.308 | 0.3634 | 0.0643 | 0.7364 | 0.0625 | 0.8516 | 0.0932 | 0.2938 | 0.858 | 0.0114 | 0.5035 | 0.2496 | 0.1129 | 0.0282 | 0.3099 | 0.0334 | 0.7639 | 0.8716 | 0.1564 |
| Allele \Locus | 225 | 226 | 227 | 228 | 229 | 230 | 231 | 232 | 233 | 234 | 235 | 236 | 237 | 238 | 239 | 240 | 241 |  |  |  |  |  |  |  |  |  |  |  |  |  |  |  |
| Allele 0 | 0.8493 | 0.9039 | 0.0206 | 0.5097 | 0.5075 | 0.5869 | 0.2693 | 0.0325 | 0.677 | 0.151 | 0.9946 | 0.9813 | 0.0857 | 0.9429 | 0.9561 | 0.9649 | 0.5623 |  |  |  |  |  |  |  |  |  |  |  |  |  |  |  |
| Allele 1 | 0.1507 | 0.0961 | 0.9794 | 0.4903 | 0.4925 | 0.4131 | 0.7307 | 0.9675 | 0.323 | 0.849 | 0.0054 | 0.0187 | 0.9143 | 0.0571 | 0.0439 | 0.0351 | 0.4377 |  |  |  |  |  |  |  |  |  |  |  |  |  |  |  |

**Note**: Allele 0, absent frequency of the bands per locus; Allele 1, present frequency of the bands per locus.

1. ^^ Correspondence author

   E-mail: [ljj@nwsuaf.edu.cn](mailto:ljj@nwsuaf.edu.cn) [↑](#footnote-ref-2)
